# Analysis of Residues in Environmental Samples

**DOI:** 10.3390/molecules28073046

**Published:** 2023-03-29

**Authors:** Ewa Szpyrka, Magdalena Słowik-Borowiec

**Affiliations:** Institute of Biotechnology, University of Rzeszow, 1 Pigonia St., 35-310 Rzeszow, Poland; mslowik@ur.edu.pl

The state of the environment is very important for our lives and for that of future generations. Human activity results in the continuous pollution of our planet with many substances that could persist in the environment, circulate between all of its elements, and pose a hazard to all living organisms. Thus, the development of advanced analytical methods for the determination of these substances with a very high sensitivity, even of trace levels in environmental samples of various types, represents a challenge and necessity of our time.

Małysiak and Kiljanek (2022) took up the very important task of developing a method for the determination of glyphosate, its metabolite aminomethylphosphonic acid (AMPA), and glufosinate ammonium residues in beebread samples. The authors used liquid chromatography coupled with tandem mass spectrometry (LC–MS/MS) as their analytical technique. The method was optimized (four clean-up methods were compared), validated, and positively verified in an international proficiency test. It was then applied to analyses of real beebread samples. The optimized method could be used for the assessment of beebread’s contamination with residues of glyphosate, its metabolite AMPA, and glufosinate ammonium [1].

Salahshoor et al. (2022) studied the performance of the fabricated molecularly imprinted polymer (MIP) sensor of atrazine and its metabolites, desethylatrazine (DEA) and desisopropylatrazine (DIA), in natural water samples obtained from seven sampling sites near Columbia, Missouri. Samplings were conducted to investigate the levels of atrazine and its metabolites during the preplant and cultivation seasons after rainfalls. Additionally, the pesticide levels measured by the sensor were verified against values obtained via the LC/MS technique. The authors stated that portable sensors can be used as the first screening tool for the presence of pesticides in situ [2].

Książek-Trela et al. (2022) evaluated the influence of commercial effective microorganisms on the degradation of two persistent herbicides, diflufenican and flurochloridone. The authors applied three formulations containing different bacteria and yeast. All of the studied EM formulations increased the degradation of flurochloridone by 19.3% to 31.2% at most. On the contrary, in the case of diflufenican all of the studied formulations prolonged its degradation due to the increased acidity of the soil environment and increased persistence of that substance at lower pH levels. Moreover, dissipation kinetic equations of diflufenican and flurochloridone, as well as the times of the half-life of these substances in laboratory conditions, were calculated [3].

Słowik-Borowiec et al. (2022) developed new analytical approaches to the simultaneous identification as well as quantification of 94 pesticides and 13 polycyclic aromatic hydrocarbons (PAHs) in 5 representative matrices (pepper, apple, lettuce, wheat, and soil) via an analysis with a GC-MS/MS system. The procedure was optimized by changing the solvent used during the extraction, as well as by reducing the amount of water during the extraction from cereals. An additional modification was the use of florisil instead of GCB in the sample clean-up step. Proposed changes allow for the shortening of the sample preparation time (by 20%) and a reduction in the consumption of organic solvents (by 17%). In order to determine the usefulness of the developed method, a validation was carried out with the parameters of linearity, recovery, precision, and limits of detection as well as quantification assessed, in addition to the measurement of uncertainty. The validation procedure was performed in accordance with the European Commission guidelines (SANTE/12682/2019) and Commission Regulation (EU) (2011) No 836/2011. The overall linearity, precision, and accuracy parameters were highly satisfactory, with the exception of six pesticides. The extended uncertainty of the method was also acceptable. Ultimately, the method was successfully applied to determine pesticide residues in commercial samples of fruits, vegetables, and grains, as well as soil samples, for PAHs [4].

Cybulski et al. (2022) evaluated the influence of water and wastewater treatment on reductions in the concentrations of zinc (Zn), nickel (Ni), iron (Fe), manganese (Mn), copper (Cu), lead (Pb), and arsenic (As). The analysis was performed with inductively coupled plasma atomic emission spectrophotometry (ICP-AES). Reductions in the most dangerous elements during water treatment fluctuated from 48.5% (As) to 97% (Pb). Wastewater treatment reduced the concentrations of analyzed elements by 28.6% to 60.8%, and of the most toxic elements (Pb and As) by over 50%. Trace element concentrations in treated wastewater were below the maximum acceptable concentration (MAC) values, and ranged from 1.15% (Pb) to 6.23% (As) of the MACs of the toxic elements. The concentrations of both the essential elements (Zn, Ni, Fe, Mn, and Cu) and the toxic elements (Pb, As) in drinking water were below the MACs. This study confirms the necessity of continuing research on the effectiveness of various water treatment methods and filtration beds, as well as that considering drinking water along with the food that humans consume when estimating sources of trace elements’ intake [5].

Kopańska et al. (2022) took up the topic of acrylamide—an organic compound that forms during thermal treatments, such as frying or baking, of carbohydrate-containing food products, particularly starches. This molecule is present in chips, crisps, coffee, cakes, and biscuits, as well as bread. Acrylamide action in an organism leads to oxidative stress, inflammation, apoptosis, and DNA damage. Kopańska et al. (2022) studied the influence of acrylamide on inflammatory processes, the oxidative stress it causes in the cholinergic system, and the possibility of reducing inflammation via supplementation with α-tocopherol. For this purpose, an *in ovo* model was used, where the embryos were exposed to acrylamide, α-tocopherol, and a cocktail of these substances. Subsequently performed biochemical assays examined the effect of the chosen substances on oxidative stress (malondialdehyde, MDA, and reduced glutathione, GSH) and acetylcholinesterase activity (AChE). The results showed that acrylamide decreased AchE activity by about 25% in comparison to the control group, and this effect was reduced by administering α-tocopherol. The concentration of MDA significantly increased in the group given acrylamide, while the observed concentration was lower in the group with α-tocopherol, when compared to the control group. The research showed a decrease in the concentration of glutathione after the administration of acrylamide; the protective effect of α-tocopherol was only slightly visible in this case [6].

In conclusion, the analysis of residues in environmental samples is a very important and broad topic, considering the enormous number of chemical substances that are used worldwide and their diversity in terms of formulae, physicochemical properties, and persistence. We need to continue analytical research and incessantly develop as well as improve modern techniques in order to maintain a clean environment and protect human health, in addition to all living organisms.
